# Co-presence of classical scrapie but not classical Bovine Spongiform Encephalopathy in transmissions from Dutch sheep with atypical scrapie

**DOI:** 10.1099/jgv.0.002202

**Published:** 2025-12-23

**Authors:** Lucien J. van Keulen, Corry H. Dolstra, Ruth Bossers-de Vries, Alex Bossers

**Affiliations:** 1 Wageningen Bioveterinary Research, Wageningen University and Research Centre, Lelystad, The Netherlands

**Keywords:** atypical scrapie (AS), classical scrapie (CS), prion, protease-resistant prion protein (PrP^res^), scrapie-associated prion protein (PrP^Sc^), transgenic mice

## Abstract

Two cases of atypical scrapie (AS) from Dutch sheep were passaged to WT mice and ovine and bovine prion protein (PrP) transgenic mice. Both AS isolates failed to transmit to WT mice but were successfully transmitted to ovine PrP transgenic Tg338 and Tg59 mice, carrying two different sheep PrP alleles. Western blot analysis of the brains from both mouse lines showed a similar protease-resistant band of ~6–8 kDa, which corresponded to the amino acids ~93–155 of the ovine PrP. Sagittal and coronal immunohistochemical profiles of the abnormal scrapie-associated PrP (PrP^Sc^) in the mouse brain were constructed and revealed that PrP^Sc^ was mainly located in white matter structures in the frontal parts of the brain. Cross-passages between the Tg338 and Tg59 mice showed that the same prion strain was isolated in each mouse line.

In the primary transmission of one of the AS isolates to Tg338 mice, two mice showed a very short incubation period, a mixed PrP^Sc^ profile and a classical scrapie banding pattern in the Western blot. Secondary transmission from these mice into WT VM mice and Tg338 mice revealed a similar PrP^Sc^ profile and incubation period to the classical murine scrapie strain 22A. Both primary and secondary transmissions of the two AS isolates to bovine PrP transgenic mice were negative, so we cannot confirm the previously reported co-presence of classical bovine spongiform encephalopathy (c-BSE) with AS.

## Introduction

Classical scrapie (CS) in sheep is a member of a group of diseases known as transmissible spongiform encephalopathies (TSEs) or prion diseases. The main feature of TSEs is the accumulation in the brain (and sometimes in lymphoid tissues) of an abnormal form of the prion protein (PrP), called scrapie-associated PrP (PrP^Sc^). PrP^Sc^ differs from its normal cellular counterpart, PrP^C^, by its relative proteinase K resistance, leaving a protease-resistant core called PrP^res^, whereas PrP^C^ is completely digested into amino acids or small peptides [1,2]. Western blots (WBs) of proteinase K-treated brain tissue from sheep with CS typically show the CS pattern consisting of three bands of PrP^res^ (due to differential glycosylation), with the unglycosylated fraction at ∼21 kDa. The incidence of CS is highly dependent on the genotype of the ovine prion protein gene (PRNP) at codons 136, 154 and 171 with animals homozygous for the VRQ allele (expressing valine (V), arginine (R) and glutamine (Q) respectively at these positions) at very high risk of disease and animals homozygous for the ARR allele (expressing alanine (A), arginine (R) and arginine (R) respectively) at the lowest risk [3,5].

CS has been known as a disease of sheep for many centuries, but atypical scrapie (AS) was more recently discovered in Norway in 1998 (Nor98) [6]. After the introduction of the active TSE surveillance system for small ruminants in the EU, AS has been reported in most EU member states and in many other countries around the world, even in countries that had never experienced cases of CS [7,9]. Almost all AS cases have been detected in elderly sheep by active surveillance and were not reported as clinical scrapie cases. A study of formalin-fixed archival brain material in the UK has traced back cases of AS to at least 1987 [10], showing that atypical/Nor98 scrapie has existed for many years without being detected .

AS not only differs from CS in its clinical presentation but also in the sheep PrP genotypes that are affected. An increased risk of AS has been identified in sheep with phenylalanine (F) instead of leucine (L) at codon 141 (termed AF_141_RQ) or histidine (H) at codon 154 (termed AL_141_HQ), No protective effect has been seen in ARR homozygous or heterozygous animals [7,11,14].

AS also differs from CS in the molecular features of PrP^res^, as proteinase K treatment not only trims the PrP^Sc^ molecule from the N-terminus but also from the C-terminus, leaving a core peptide spanning ~aa 93–155 (based on the ovine PrP sequence) and a single PrP^res^ band of ~7–12 kDa without glycosylation sites [6,15, 16]. In AS, PrP^res^ accumulates especially in the cerebellum and the more rostral areas of the brain, contrary to CS, where PrP^res^ is more pronounced in the brainstem [17]. The brainstem, which is normally used in the EU surveillance protocols for CS, is therefore considered a very poor sampling area for AS with regard to diagnostic sensitivity [18,19]. PrP^res^ is not detected in the lymphoreticular system in AS, contrary to CS, so pre-clinical diagnosis of AS based on the detection of PrP^Sc^ outside the central nervous system is not possible [18].

Natural transmission of AS under field conditions is thought to be unlikely or not to occur at all, although experimental transmission of AS in sheep via the oral route is successful (albeit only in neonatal lambs) [20,22]. This is also related to the fact that no PrP^res^ has been detected in the lymphoreticular system, making shedding of PrP^res^ into the environment unlikely. It has therefore been postulated that AS is a ‘spontaneous’ occurring disease in elderly sheep, although the term ‘idiopathic’ disease may be more suitable until any evidence of an acquired infection has been completely ruled out.

In contrast to CS, AS cannot be transmitted to WT mice, but transmissions to bank voles and transgenic mouse models have been successful [18]. In the Netherlands, 18 cases of AS have been identified so far through the active scrapie surveillance system starting in 2001. We passaged the first two Dutch AS isolates to WT and both ovine and bovine PrP transgenic mice and studied in detail the WB PrP^res^ pattern and immunohistochemical PrP^Sc^ profile in the brain of these mice. In addition, we performed cross-transmission studies to see if the prion strain identified in each mouse line was the same or whether multiple prion strains exist in AS.

## Methods

### Mouse lines

All mouse lines were bred in-house and were originally founded by breeding couples that were kindly donated by colleagues from other research institutes (see Acknowledgements). Non-transgenic WT mice included the RIII mice expressing on the mouse prion protein gene (Prnp) the Prnp^a^ allele, encoding for leucine at position 108 (108L) and threonine at position 189 (189T), and VM mice expressing the Prnp^b^ allele, encoding for phenylalanine at position 108 (108F) and valine at position 189 (189V) on the mouse PrP gene [23,24].

Transgenic mice expressing the ovine or bovine PrP allele were established under a mouse prion protein knock-out background (Prnp^0/0^) by random integration. Tg338 mice express ~eightfold the V_136_R_154_Q_171_ allele of the ovine PrP, Tg59 mice express ~threefold the A_136_R_154_Q_171_ allele of the ovine PrP and Tg110 mice express ~eightfold the bovine PrP allele compared to PrP^C^ levels in the natural host [25,27].

### AS isolates

AS1 was the first AS case in the Netherlands detected by active surveillance in 2005. It was a Black Blazed (Dutch: ‘Zwartbles’) sheep in the category ‘fallen stock’ and carried the ALHQ/ALHQ genotype (amino acids coded at codons 136, 141, 154 and 171). AS2 was the second atypical case of scrapie that was also detected in 2005, but was in the category ‘healthy slaughter’ animals and was of the AFRQ/AFRQ genotype. Both AS cases tested positive with the WB Prionics test on brainstem material collected at the rendering plant and slaughterhouse and were confirmed as AS by PrP^Sc^ immunohistochemistry (IHC). After confirmation, the complete heads of the sheep were sent to the Dutch TSE reference institute Wageningen Bioveterinary Research (WBVR), where the cerebellum, palatine tonsils and retropharyngeal lymph nodes could be collected aseptically for transmission studies. The lymphoid tissues of both AS cases tested negative for PrP^Sc^ with IHC. Based on the evaluation of the teeth, both sheep were at least >5 years, but the exact age was unknown.

### Murine scrapie reference strain 22A

The murine scrapie reference strain 22A was obtained from the TSE Resource Centre, currently part of the Roslin Institute, University of Edinburgh, UK (Catalogue No. RC 008). It was originally isolated from Cheviot sheep inoculated with the experimental Sheep Scrapie Brain Pool (SSBP/1) and had been passaged 21 times in Cheviot sheep [28]. It was then passaged and cloned by three consecutive high-dilution, intracerebral (i.c.) passages in VM mice. We received a 10% (w/v) mouse brain homogenate from the tenth i.c. passage in VM mice and diluted it into a 1% brain homogenate to be used for our mouse inoculations.

### Mouse inoculations

For mouse passage, a 10% w/v (primary passage of ovine cerebellum or tonsil) or 1% w/v (second and higher passages of the sagittal half of mouse brain) homogenate in physiological saline containing 1.4 mg ml^−1^ ampicillin was produced with an Ultra-Turrax^®^ T25 homogenizer (IKA, Germany). After each use, the dispersing rotor of the homogenizer was immersed in 2M sodium hydroxide for at least 24 h and then autoclaved at 134 ˚C and 3 bar for 30 min. The rotor was then dismantled, and all separate parts were again immersed in 2M sodium hydroxide for a minimum of 24 h. After rinsing and assembly, the rotor was separately sealed in an autoclave bag and autoclaved for a second time at 134 ˚C/3 bar for 30 min to ensure absolute prion sterility. All inocula were made independently, with several years in between the preparation of AS1, AS2 and the murine reference strain 22A. Negative control brain inocula were prepared in an identical way, but no TSE cross-contamination was ever detected in the mouse bio-assay.

For each subpassage from a positive transmission, the brain of the clinically and PrP^Sc^-positive mouse with the shortest incubation period (IP) from the previous passage was used to prepare the homogenate, unless there were indications of the existence of multiple strains, in which case each strain was passaged separately. In the case of a negative primary transmission, further subpassage was performed by pooling the brains of all mice from the negative primary transmission into a 10% (w/v) homogenate. After each homogenate had been routinely checked for bacteriological sterility, 0.02 ml was inoculated under general anaesthesia into the left cerebral hemisphere of randomized groups of 5–25 RIII, VM, Tg338, Tg59 or Tg110 mice. Subpassages in mice were called homologous when they were performed in mice with the same PrP genotype as the mouse used for the inoculum. In case the PrP genotypes differed, the term heterologous or cross-passage is used.

### Calculation of IP and attack rate

Inoculated mice were assigned a group/cage number and were monitored blindly each day for clinical disease. Mice were euthanized at terminal disease, and the IP was defined as the number of days between inoculation and terminal end-point/death. Mean IP±sd (in days) was calculated for all groups of mice. Attack rates (ARs) were calculated as the number of mice (*n*) that were clinically and PrP^Sc^ positive (clin+/PrP^Sc^+) divided by the number of mice (*N*) still alive at the time of death of the first clin+/PrP^Sc^+ mouse. Animals that were clin−/PrP^Sc^+ were considered to be pre-clinically infected and were excluded from the calculations of the mean IP and AR. In the case of a negative transmission, the survival time of the last surviving mouse is given to indicate the maximum life span, and the AR is shown as 0/total number of mice injected. Bio-assays were ended at 900 days post-inoculation when no PrP^Sc^+ mice had been found.

### Necropsy and histological procedures

Mice that were found dead were decapitated and the heads frozen at −20 ˚C until further processing. The frozen brain was collected using TSE-sterile instruments and cut sagittally. One half of the brain was fixed in 10% neutral-buffered formalin for 24 h, while the other half was stored at −20 ˚C for sub-passage or WB analysis.

Mice that were at the endpoint of terminal disease were euthanized by cervical dislocation and decapitated. The top of the skull was carefully removed, and the head was either frozen at −20^◦^C (and processed as described above for the mice that were found dead) or fixed in 10% phosphate-buffered formalin for at least 24 h. After formalin fixation, the brain was removed from the skull and cut into the following coronal levels: (A) septal nuclei, corpus striatum and anterior cortex; (B) diencephalon (thalamus and hypothalamus), hippocampus, lateral and entorhinal cortex; (C) mesencephalon, pons and occipital cerebral cortex and (D) pons and anterior cerebellum.

Formalin-fixed coronal and sagittal brain halves were fixed for another 24 h before routine processing into paraffin blocks. Paraffin blocks were cut into 4 µm thick sections that were collected on aminopropyltriethoxysilane-coated glass slides (Sigma, USA) and dried for at least 48 h in a 37 ˚C incubator.

### Antibodies

PrP-specific monoclonal antibodies that were used included: 12B2 (_93_WGQGG_97_), 9A2 (_102_WNK_104_), 6C2 (_114_HVAGAAA_120_) (all by WBVR, the Netherlands), Sha31 (_148_YEDRYYRE_155_) (Bio-Rad, France), SAF84 (_166_YRPVDQY_172_) (Bertin Pharma, France) and R145 (_213_VEQMCITQYQR_223_) (rat monoclonal, APHA, UK). Corresponding epitopes are based on sheep PrP amino acid numbering as mapped by Pepscan analysis using 15-mer solid-phase synthetic peptides [29].

### WB analysis and PrP^res^ profiling

Longitudinally cut mouse brain halves were homogenized to 10% (w/v) tissue homogenates in lysis buffer using disposable polypropylene pestles as described previously [30]. Cerebellum from the ovine positive-control AS sheep was homogenized to a 25% (w/v) homogenate. Brain homogenates were digested with 5.5 µg of Prot K (Merck) per 50 ul homogenate for 60 min at 37 °C. After digestion, Prot K was inactivated with 10 ul Pefabloc 3 mg ml^−1^ (Roche). Samples were precipitated with 1-propanol and centrifuged for 5 min at 18.000***g***, and the pellets were resuspended in NuPAGE LDS sample buffer (Thermo Fisher Scientific) with Orange G (Sigma). Samples were heated for 10 min at 96 °C, and 0.97–1.65 mg tissue equivalent was loaded onto SDS-PAGE gels together with the molecular mass marker SeeBlue (Invitrogen). For electrophoresis, 17-well NuPAGE 12% Bis-Tris 1.0 mm precast gels were run in triplicate in 2-(N-morpholino)ethanesulfonic acid (MES) buffer at 100 V for 80 min. The three gels were electrotransferred simultaneously onto polyvinylidene difluoride (PVDF) membranes (Immobilon-FL, Millipore) with 20% methanol transfer buffer (Novex) for 30 min at 50 V, followed by 45 min at 150 V. After transfer, the blots were blocked overnight at 4 °C in Tris Buffered Saline Tween 20 (TBST) with 5% casein. The blots were incubated with three different antibodies: 12B2, 9A2 and SAF84 at respective concentrations of 0.2, 0.5 and 0.5 µg IgG per millilitre in TBST with 5% casein for 60 min at room temperature (RT). After incubation, the membranes were washed three times for 5 min with TBST. Rabbit anti-mouse-AP (DAKO D0314) was used as the secondary antibody at a concentration of 0.46 µg IgG per millilitre in TBST with 5% casein for 60 min at RT. Membranes were washed three times for 5 min with TBST and for an additional 5 min with UltraPure distilled water. BCIP/NBT (5-bromo-4-chloro-3-indolyl-phosphate/ nitro blue tetrazolium) liquid substrate (Sigma) was used to develop the blot, and after 5 min, the reaction was stopped by draining the blot and subsequent washing with tap water. Blots were scanned with the Amersham 600 Imager, and molecular weights were determined with the software ImageQuant 1D gel analysis.

### IHC and PrP^Sc^ profiling

Sections were deparaffinized in xylene, rehydrated in graded alcohols and pretreated by immersion in formic acid for 10 min, followed by autoclaving at 121 ˚C for 5 min in citrate buffer pH 6.0 (Antigen Unmasking Solution, Vector Laboratories). After cooling down with running tap water, sections were incubated for 60 min with the mouse monoclonal antibody 6C2 for the WT mice or the rat monoclonal antibody R145 for the transgenic mice (both at 0.4 µg IgG per millilitre). A horseradish peroxidase (HRP) anti-mouse IgG polymer (SuperBoost, Invitrogen) or anti-rat IgG polymer (ImmPRESS, Vector Laboratories) was used, respectively, as the secondary antibody, and colour was developed with diaminobenzidine (DAB+) substrate (Dako, Agilent). DAB colour was intensified by 20 min immersion in 0.5% copper sulfate (CuSO_4_) solution (Merck). Sections were briefly counterstained with haematoxylin, dehydrated in graded alcohols and mounted permanently.

Stained sections were scanned with a BX51 microscope equipped with a motorized stage and Cell Sense^®^ imaging software (Olympus, Germany) as described previously. True colour IHC images were phase colour coded according to the intensity of the DAB immunolabelling, with dark brown pixels (intensity, 1–60) displayed in dark red, middle brown pixels (intensity, 61–130) in red and light brown pixels (intensity, 131~190) in yellow.

### Statistical analysis

Statistical analysis of IPs was performed using GraphPad Prism 10 (GraphPad Software Inc). Differences between two groups were considered statistically significant if the probability for equality was <0.05 in Welch’s t-test (adapted Student’s t-test for unequal sample size and variance).

## RESULTS

### Homologous subpassage of AS

#### Transmission

AS1 and AS2 did not transmit to WT RIII and VM mice or to bovine transgenic Tg110 mice either at primary passage or at secondary passage (Table 1). However, both AS1 and AS2 transmitted efficiently to the ovine PrP transgenic mice with 100% ARs. In the ovine ARQ transgenic Tg59 mice, the IPs to terminal end point/death did not seem to stabilize even at the third passage (fourth passage underway). In the ovine VRQ transgenic Tg338 mice, the IP stabilized at 239±5 days for AS1 and at 238±8 days for AS2, which is not significantly different (*P*=0.82). In the primary transmission of AS1 in Tg338 mice, two mice died with clinical signs at 110 days post-inoculation, months before the rest of the inoculated mice. The brains of these two mice showed a different PrP^res^ and PrP^Sc^ profile than the other mice, so they were passaged separately (referred to as AS1* in the following sections). No transmission was seen when tonsillar tissue of AS1 and AS2 was inoculated into WT RIII or ovine VRQ transgenic Tg338 mice.

**Table 1.** IPs and ARs of the homologous passages of AS1 and AS2 in the various mouse lines

		AS1 (ALHQ/ALHQ)				AS2 (AFRQ/AFRQ)			
		Brain		Tonsil		Brain		Tonsil	
	P#	IP	AR (*n*/*N*)	IP	AR	IP	AR (*n*/*N*)	IP	AR (*n*/*N*)
**Tg59**	1	551±90	10/10			455±52	10/10		
	2	464±135	8/8			449±59	9/9		
	3	579±78	8/8			549±38	9/9		
**Tg338**	1	212±19^*^	23/25	*>900*	*0/13*	263±13	15/15	*>900*	*0/15*
	2	263±26	13/13			267±11	10/10		
	3	260±19	15/15			244±8	5/5		
	4	239±5^†^	4/4			238±8^†^	5/5		
**Tg110**	1	*>880*	*0/15*			*>836*	*0/15*		
	2	*>829* ^‡^	*0/15*			*>569* ^‡^	*0/9*		
**RIII**	1	*>900*	*0/25*	*>900*	*0/8*	*>900*	*0/9*	*>900*	*0/15*
**VM**	1	*>825*	*0/25*			*>834*	*0/10*		

*Two of 25 mice died at 110 days post-inoculation (clin+/PrPSc+), which is a significantly shorter IP than the rest of the mice (23/25 mice at 212±19 days, *P*<0.0001 in Welch’s t-test). This subgroup is further referred to in the text as AS1* and is not included in the calculation of the IP of AS1.

† IPs not significantly different (*P*=0.82, Welch’s t-test).

‡Blind passage of a brain pool from the mice in the primary passage.

Negative passages are dispayed in italics, blank spaces mean that the passage is not done in this mouse line

AR, Attack rate (see Methods); IP, incubation period to terminal end point/death (mean±sd in days); P#, Passage number.

#### PrP^res^ analysis

**Fig. 1.**
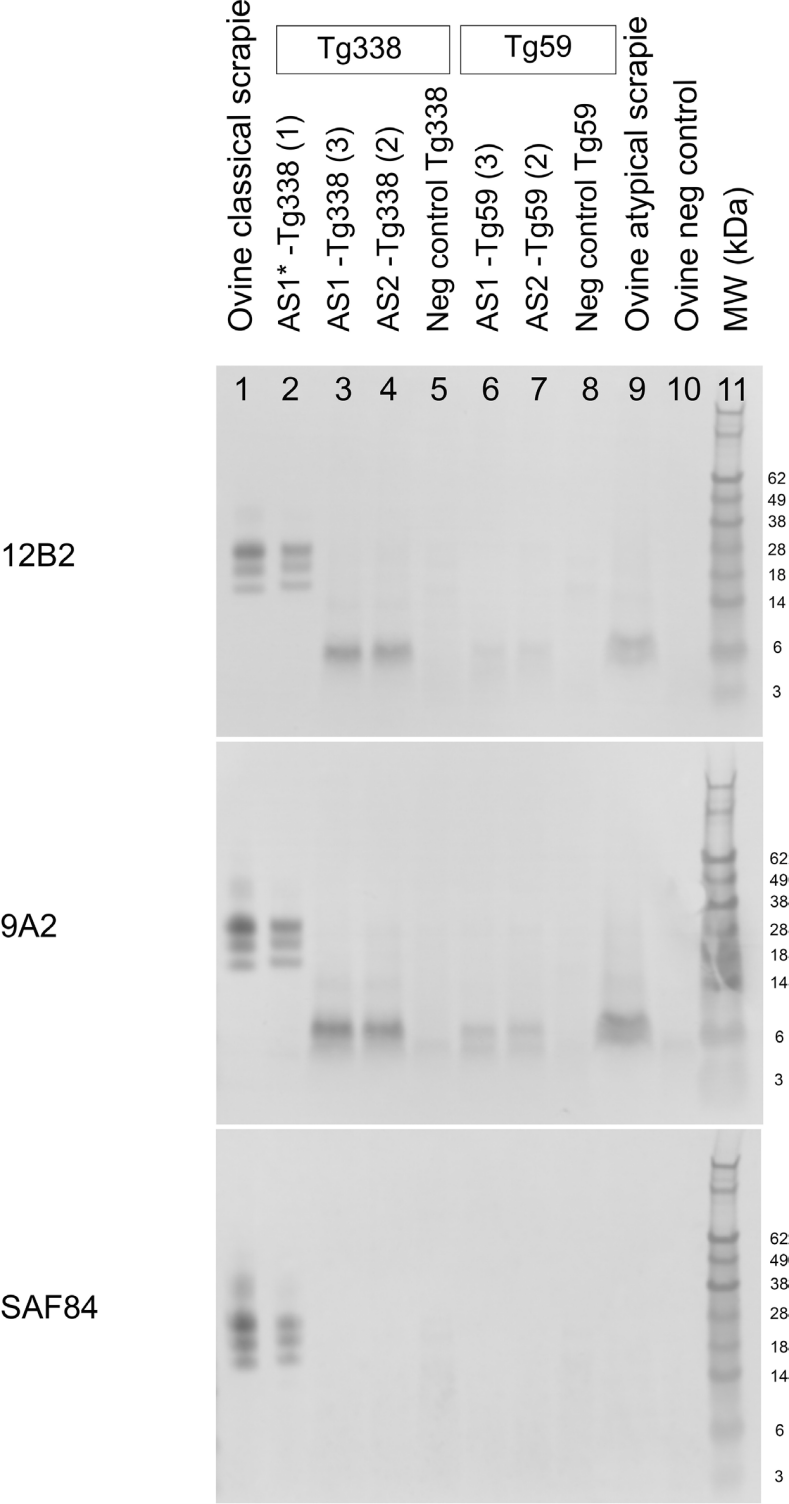
WB analysis of the brain tissue from ovine PrP transgenic mice inoculated with AS1*, AS1 and AS2, using three different monoclonal antibodies to ovine PrP: 12B2 (epitope aa 93–97), 9A2 (aa 102–104) and SAF84 (aa 166–172). AS1* in Tg338 mice (lane 2) shows the typical PrP^res^ triplet banding in all three blots, similar to ovine CS (lane 1). AS1 and AS2 from ovine transgenic mice (lanes 3, 4, 6 and 7) show a 6–8 kDa band in the 12B2 and 9A2 blots, similar to ovine AS (lane 9), which is not present in the SAF84 blot. A faint 4–5 kDa band is present, that is also visible in the non-inoculated mice (lanes 5 and 8), which were used as negative (neg) controls. Lane 10 shows a negative (neg) control sheep, and lane 11 contains the molecular weight (MW) marker (in kDa).

**Fig. 2.**
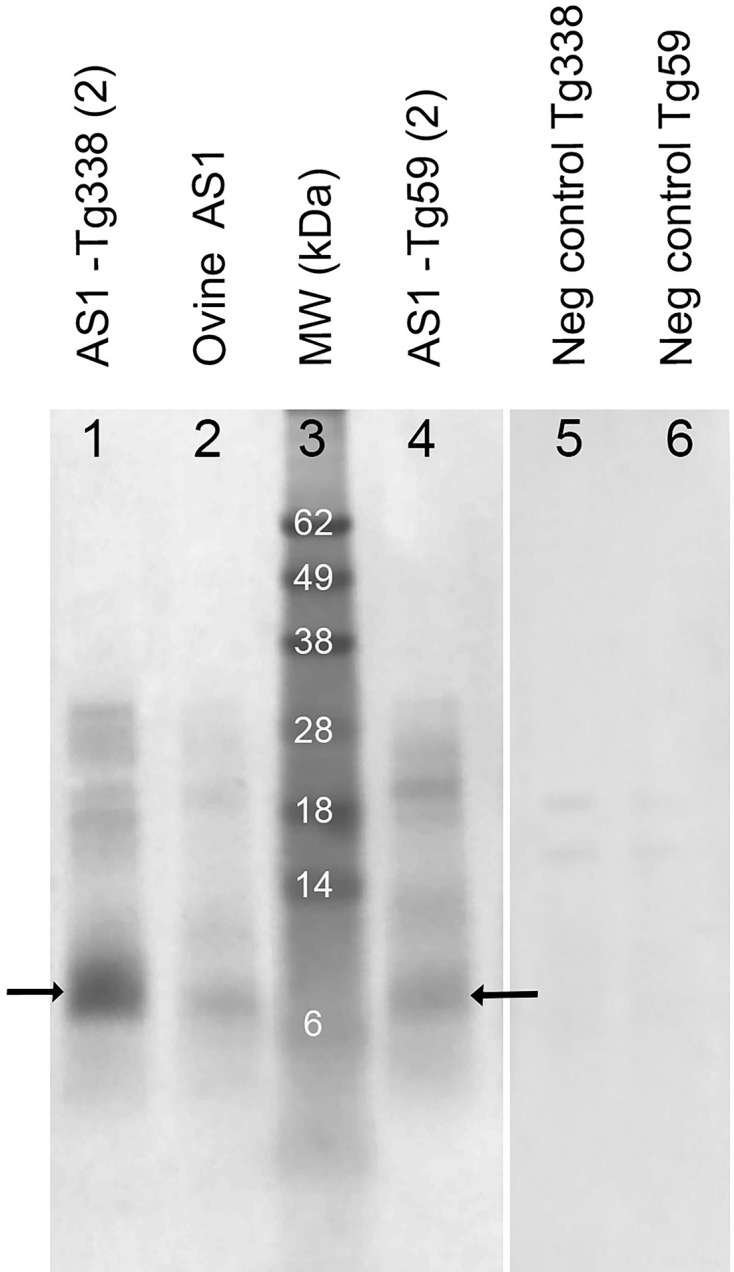
WB analysis of ovine AS1 and mouse-passaged AS1 using the monoclonal antibody Sha 31 (epitope aa 148–155). The 6–8 kDa band of PrP^res^ (arrows) is visible in the original sheep brain sample of AS1 (lane 2), as well as in the Tg338 and Tg59 mouse brain samples from the secondary passage of AS1 (lanes 1 and 4, respectively). Negative (neg) control brains from a Tg338 and a Tg59 mouse are shown in lanes 5 and 6, and the molecular weight (MW) marker (in kDa) is shown in lane 3.

WB analysis of AS1 and AS2 transmitted to ovine VRQ transgenic Tg338 mice and ovine ARQ transgenic Tg59 mice showed a 6–8 kDa band in the 12B2 (_93_WGQGG_97_) and 9A2 (_102_WNK_104_) blots, similar to ovine AS, which was not detected with the SAF84 antibody (_166_YRPVDQY_172_), indicating a C-terminal-truncated PrP^res^ fragment (Fig. 1). Additional WB analysis using the Sha31 antibody (_148_YEDRYYRE_155_) showed that Sha31 did recognize the 6–8 kDa band, placing the C-terminal end of the PrP^res^ fragment in AS between aa 155 and ~aa 166–172 (Fig. 2). However, the two Tg338 mice that died early at 110 days post inoculation (referred to as AS1*) displayed a completely different PrP^res^ profile that was similar to the typical PrP^res^ triplet banding in ovine CS (Fig. 1).

#### PrP^Sc^ profiling

**Fig. 3.**
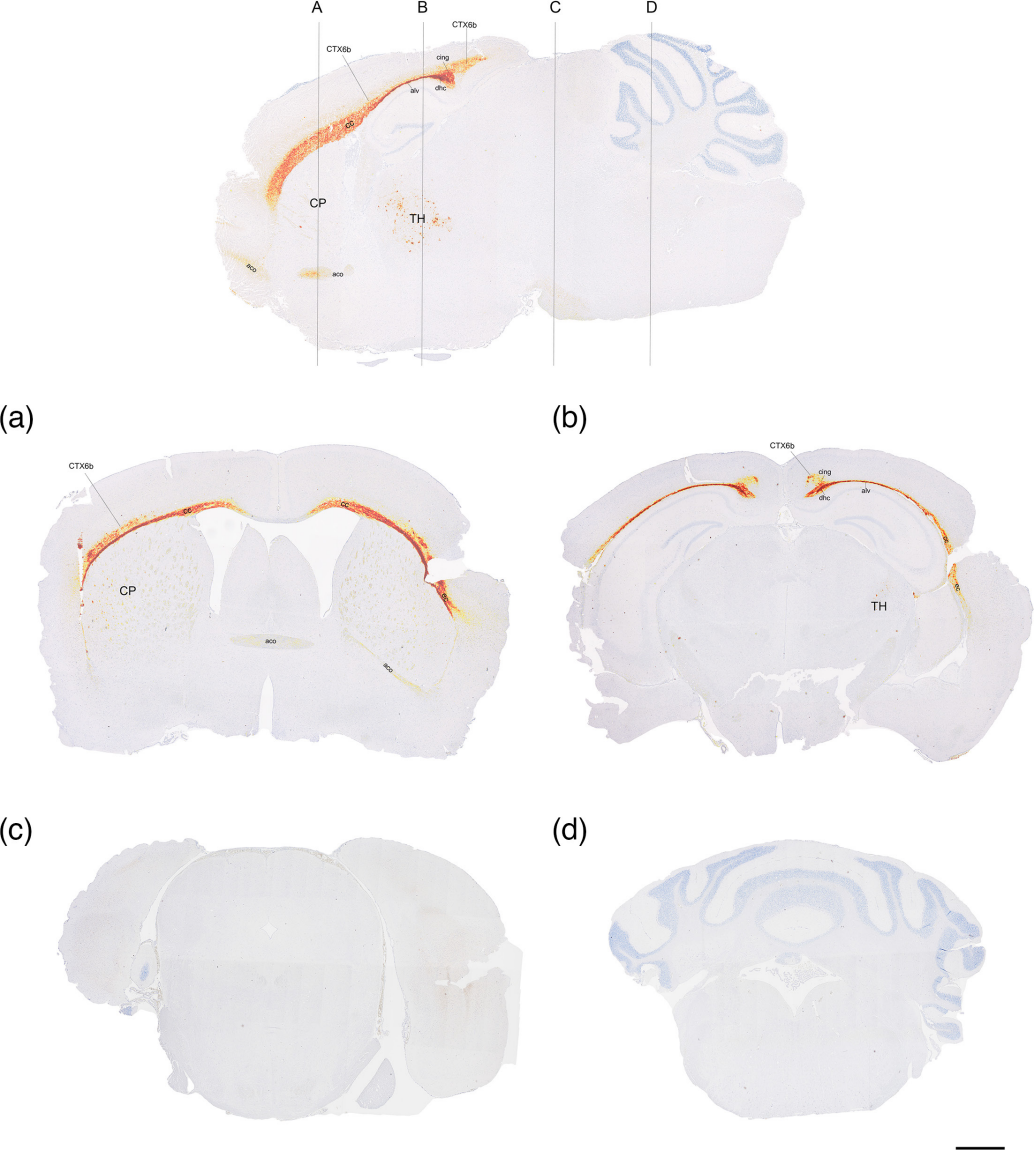
PrP^Sc^ profile of AS in a sagittal section (top) and cross-sections (**a–d**) of the brain of Tg59 mice. The PrP^Sc^ staining pattern is most intense in: (A) cc/ec, aco, CTX6b and the corticostriatal fibres of the CP; (B) cc/ec, CTX6b, dhc/alv, cing and the ventral and dorsolateral TH; and (C and D) no PrP^Sc^ detected. Inoculum from AFRQ/AFRQ sheep with AS. Bar=1,000 µm. TH, thalamus.

Immunohistochemical staining of PrP^Sc^ in the brain of Tg59 mice showed a predominant accumulation in the white matter areas of the anterior part of the brain (Fig. 3). PrP^Sc^ depositions were granular to coalescing and plaques-like, and mainly located in the corpus callosum (cc), external capsule (ec), the corticostriatal fibres of the caudoputamen (CP), the anterior commissure (aco), dorsal hippocampal commissure (dhc), alveus (alv) and the cingulum (cing). In addition, deposits were seen in the thalamus and in the cerebral cortex layer 6b (CTX6b). The PrP^Sc^ staining in the cc/ec and CTX6b was sometimes seen to extend into the temporal and occipital cerebral lobes, but no immunostaining was seen in the midbrain, cerebellum or medulla oblongata.

**Fig. 4.**
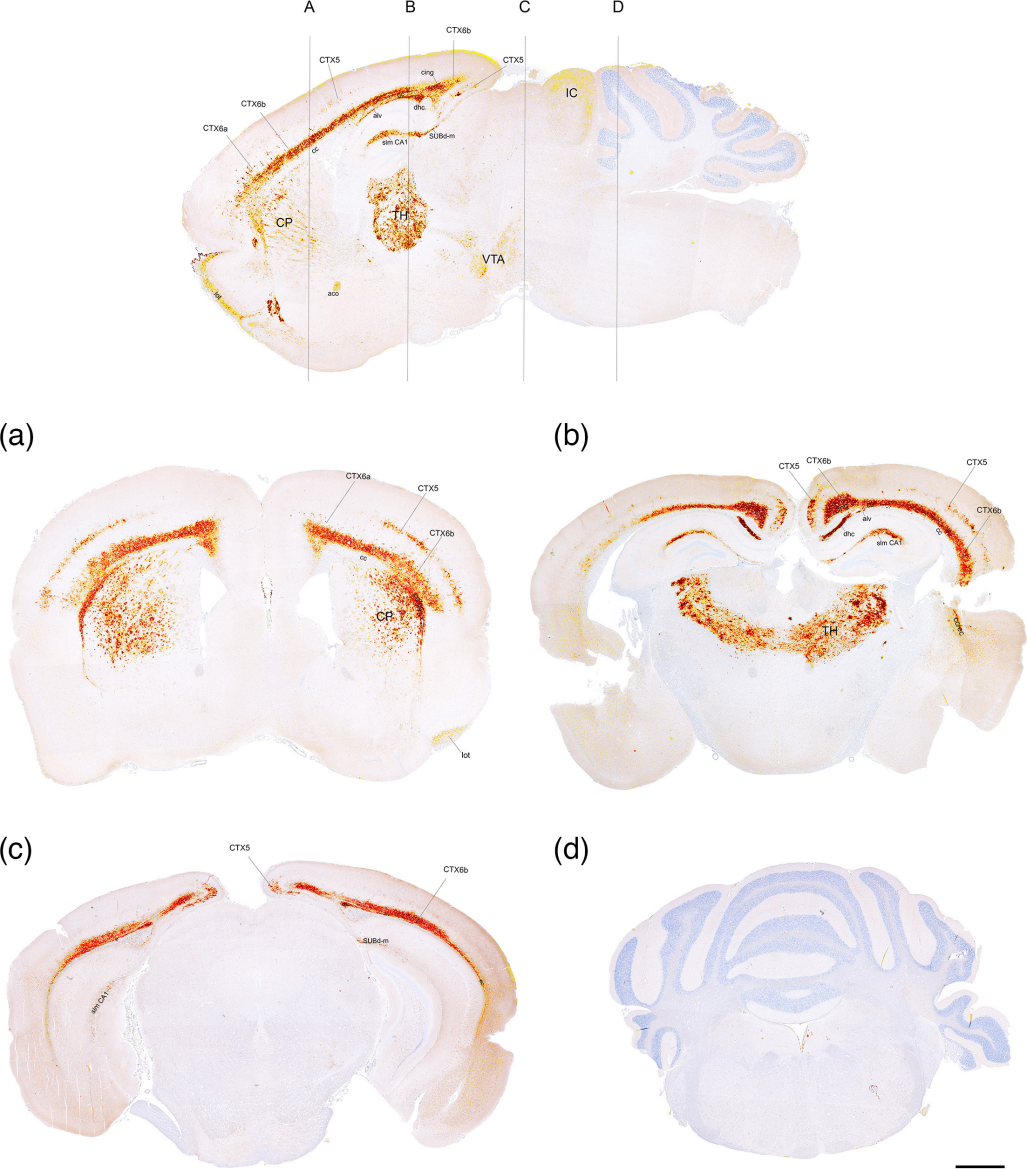
PrP^Sc^ profile of AS in a sagittal section (top) and cross-sections (**a–d**) of the brain of Tg338 mice. PrP^Sc^ staining pattern is most intense in (A) cc/ec; CTX 5, 6a and 6b; the corticostriatal fibres of the CP and the lateral olfactory tract (lot); (B) cc/ec, CTX 5 and 6b, dhc/alv, cing, slm CA1 and the ventral and dorsolateral TH; (C) ec, CTX 5 and 6b, dhc/alv and Subd-m; and (D) no PrP^Sc^ detected. In the sagittal section (top), additional PrP^Sc^ staining is seen in the aco, VTA and IC. Inoculum from AFRQ/AFRQ sheep with AS. Bar=1,000 µm. IC, inferior colliculus; slm CA1, stratum lacunosum moleculare of the CA1; Subd-m, molecular layer of the dorsal subiculum; TH, thalamus; VTA, ventral tegmental area.

In the Tg338 brain, similar PrP^Sc^ immunostaining was seen as in the Tg59 mice but with much heavier involvement of the corticostriatal fibres, CTX6b and the thalamus (Fig. 4). In addition, CTX 5 and 6a were affected, and in the hippocampus, a pronounced staining of the stratum lacunosum-moleculare of the CA1 region and the stratum moleculare of the dorsal part of the subiculum was seen. More often than in the Tg59 mice, this staining extended posteriorly into the temporal and occipital cerebral lobes and the caudal parts of the hippocampus, but no immunostaining was seen in the midbrain, cerebellum or medulla oblongata, like in the Tg59 mice.

### Heterologous subpassage of AS

**Fig. 5.**
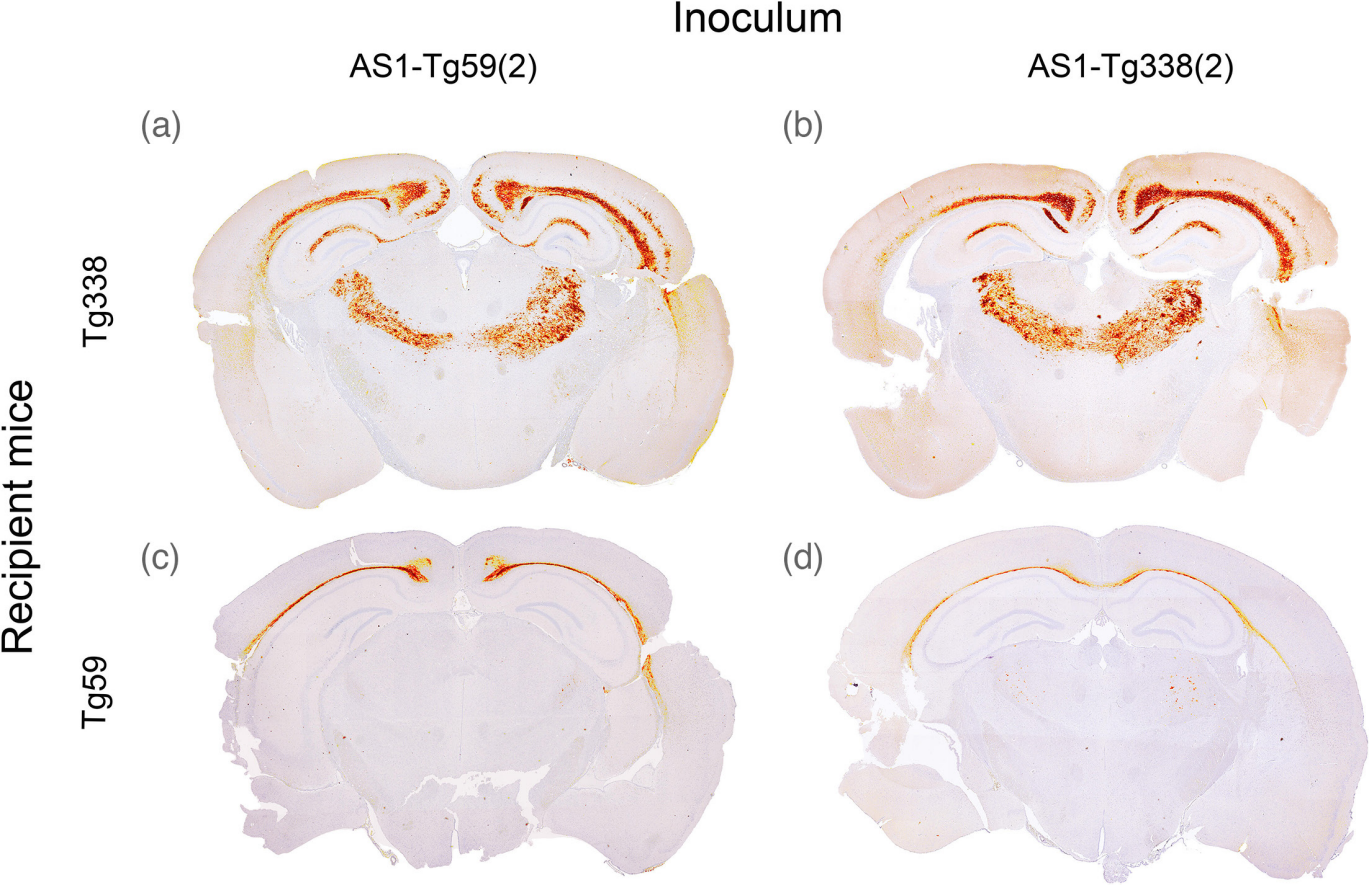
PrP^Sc^ profiles of the cross-passage of AS1 between Tg338 and Tg59 mice at the level of the thalamus. The PrP^Sc^ profile of AS1-Tg59(2) cross-passaged to Tg338 mice (**a**) is similar to the PrP^Sc^ profile of the homologous passage in Tg338 mice (**b**). The PrP^Sc^ profile of the homologous passage of AS1 in Tg59 mice (**c**) is similar to the profile of the cross-passage of AS1-Tg338(2) into Tg59 mice (**d**).

To find out if the same prion strain had been isolated in both mouse lines, cross-passages were set up to inoculate the AS1 strain from Tg338 into Tg59 and vice versa. AS1 from the second passage of Tg338 [referred to as AS1-Tg338(2)] inoculated into Tg59 showed a 100% AR but did not stabilize at further passages, similar to the homologous passage in Tg59. AS1 from the second passage in Tg59 [AS1-Tg59(2)] passed into Tg338 also showed a 100% AR with an IP of 241±21 days, which is similar to the IP of the homologous passage in Tg338 (Table 2). The PrP^Sc^ profile of the cross-transmissions between AS1 in the Tg338 and Tg59 was identical to the profiles obtained from the homologous passage (Fig. 5). AS1 passaged through either Tg338 or Tg59 failed to transmit to WT or bovine PrP transgenic mice (Table 2).

**Table 2.** IPs and ARs of the cross-passages of Tg338- and Tg59-passaged AS1 in the various mouse lines

		AS1-Tg338(2)		AS1-Tg59(2)	
	P#	IP	AR (*n*/*N*)	IP	AR (*n*/*N*)
**Tg59**	1	555±105	(14/14)		
	2	464±107	(7/7)		
	3	553±55	(10/10)		
**Tg338**	1			256±16	(13/13)
	2			241±21^*^	(9/9)
**Tg110**	1	*>900*	(*0/15*)	*>872*	(*0/15*)
	2	*>900* ^†^	(*0/14*)		
**RIII**	1	*>900*	(*0/19*)	*>810*	(*0/14*)
**VM**	1	*>900*	(*0/20*)	*>754*	(*0/15*)

*IP not significantly different from the homologous passage of AS1 in Tg338 (*P*=0.82, Welch’s t-test).

†Blind passage of a brain pool from the mice in the primary passage.

Negative passages are dispayed in italics, blank spaces mean that the passage is not done in this mouse line

AR, Attack rate (see Methods); IP, incubation period to terminal end point/death (mean±sd in days); P#, Passage number.

### Profiling and subpassage of AS1*

**Fig. 6.**
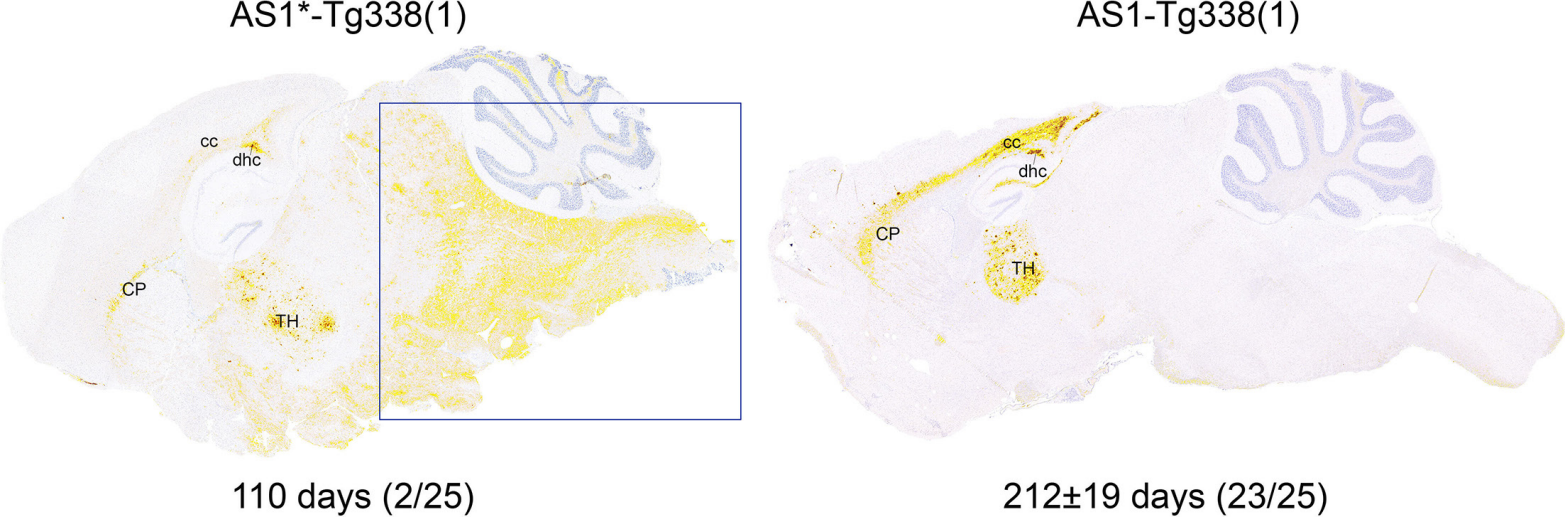
Sagittal PrP^Sc^ profile of the primary passage of AS1* and AS in the Tg338 mice. The PrP^Sc^ profile of AS1* (left) shows an early accumulation of PrP^Sc^ in the TH, CP and dhc, which are the predilection sites for PrP^Sc^ accumulation of AS in the Tg338 mice (right). However, AS1* also shows PrP^Sc^ accumulations in the posterior parts of the brain, e.g. midbrain, pons and medulla oblongata (frame), which are lacking in AS. IPs and ARs are shown at the bottom. TH, thalamus.

Transmission of AS1 to Tg338 mice resulted in 2/25 mice that died at 110 days post inoculation, which was much shorter than the IP of the other 23 inoculated mice (212±19 days; Table 1). We therefore designated this subgroup AS1* to differentiate them from the other mice. The PrP^res^ WB profile of AS1* was different from the AS profile and similar to that of CS (Fig. 1). In addition, the mice in AS1* also showed a different immunohistochemical PrP^Sc^ profile (Fig. 6). Their PrP^Sc^ profile had some characteristics of the AS PrP^Sc^ profile, with PrP^Sc^ deposits in the thalamus, CP and dhc, but these were less pronounced (bearing in mind that these mice died approximately halfway through the IP of the other mice in the AS1 group). However, contrary to the AS1 group, there were additional PrP^Sc^ deposits in the posterior parts of the brain, especially the pons and medulla oblongata, which showed a different staining pattern with a fine granular structure with minimal coalescing of PrP^Sc^ (Fig. S1, available in the online Supplementary Material).

Since the IP, PrP^res^ and PrP^Sc^ profiles of AS1* differed from the other mice in the group, indicating the presence of a second prion strain, we subpassaged the brains of these mice separately to identify this other prion strain. Subpassage of AS1* in Tg338 mice resulted in a very short IP of 70±3 days, which was similar to the IP of the murine scrapie reference strain 22A in Tg338 mice (71±3 days; Table 3).

**Table 3.** IPs and ARs of the passages of ovine AS1* and the murine reference strain 22A in Tg338 mice and VM mice

		AS1*		22**A**^†^	
	P#	IP	AR (*n*/*N*)	IP	AR **(*n*/*N*)**
**Tg338**	1	110	2/23	665±156	8/16
	2	73±1	15/15	79±4	20/20
	3	70±3^‡^	4/4	71±3^‡^	4/4
**VM**	1	576±63	11/20	218±5^§^	20/20
	2	262±14	15/15		
	3	221±2^§^	10/10		

†Passaged and cloned in VM mice.

‡IPs not significantly different (*P*=0.81, Welch’s t-test).

§IPs not significantly different (*P*=0.20, Welch’s t-test).

blank spaces mean that further subpassage is not done in this mouse line

AR, Attack rate (see Methods); IP, incubation period to terminal end point/death (mean±sd in days); P#, Passage number.

**Fig. 7.**
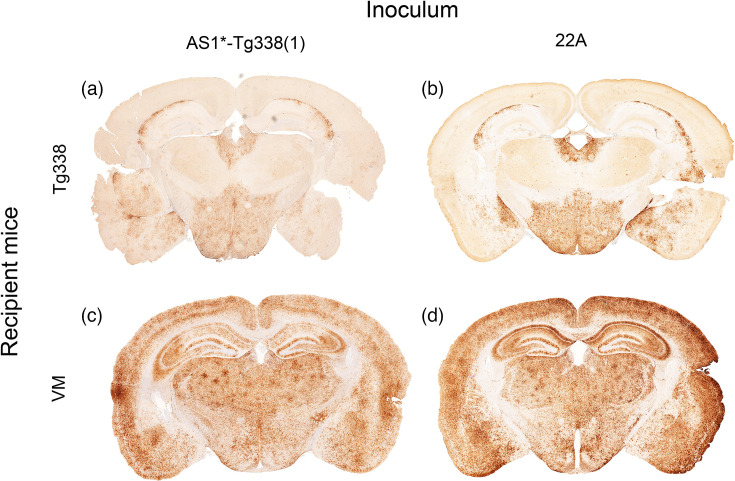
Comparison of the PrP^Sc^ profile of the subpassages of AS1* and the murine reference strain 22A in Tg338 (top) and VM mice (bottom). The profile of AS1* subpassaged in Tg338 mice (**a**) is similar to the profile of 22A in Tg338 mice (**b**). In addition, subpassage of AS1* in VM mice (**c**) results in an identical PrP^Sc^ profile to that of 22A in VM mice (**d**).

In addition, the PrP^Sc^ profile of AS1* in Tg338 mice was similar to the profile of murine 22A in Tg338 mice (Fig. 7a, b). Since the murine scrapie reference strain 22A had originally been isolated in VM mice, we also transmitted AS1* to VM mice. The IP of AS1* in the VM mice stabilized after three passages at 221±2 days, which is not significantly different from the IP of 218±5 days of murine 22A in VM mice (Table 3). Comparison of the PrP^Sc^ profiles of AS1* and murine 22A in VM mice also showed that these were identical (Fig. 7c, d).

## Discussion

We inoculated brain homogenates from two Dutch AS sheep into WT as well as ovine and bovine PrP transgenic mice. No transmission was seen in the WT mice, which is in accordance with previous reports [31,33]. However, transmission of AS to ovine transgenic mice was successful, as has been reported previously for ovine VRQ transgenic mice, as well as for ovine ARQ transgenic mice [13,31,33]. In these studies, there is some variety regarding the estimated molecular weight of the PrP^res^ band in the brain of the ovine transgenic mice with AS, ranging from 5 to 12 kDa, which is most likely due to inter-laboratory differences in electrophoretic and proteolytic conditions. However, based on epitope recognition by various antibodies, there is general agreement on the amino acid length of the PrP^res^ fragment in transgenic mice with AS. In all studies, the P4 and/or 12B2 antibody recognized this PrP^res^ fragment, which means that it contains at least the aa 93–97 epitope at the N-terminus. In addition, this fragment is also recognized in all studies using antibodies L42 and/or Sha31, so the PrP^res^ fragment expands C-terminally to at least aa 148–155. The minimal length of the PrP^res^ fragment in AS would therefore extend from aa 93 to aa 155, which should make the theoretical weight, when calculated from the 63 individual amino acid residue weights, to be around 7 kDa. Few studies report on the use of antibodies recognizing epitopes further C-terminally of aa 155. We have previously shown that the antibody SAF84 does not recognize the PrP^res^ fragment in sheep with AS [15]. In this report, we show that the same holds true for the PrP^res^ fragment in ovine PrP transgenic mice with AS. Therefore, both in sheep and in ovine PrP transgenic mice with AS, the PrP^res^ fragment is lacking (part of) the SAF84 epitope aa 166–172. It could, however, still have a ragged C-terminal end, thus extending the protease-resistant fragment to ~aa 166–172 and the calculated weight to ~9 kDa.

WBs and histopathology of AS in sheep can also vary between the different isolates in each country. Transmissions of AS to ovine PrP transgenic mice seem to suggest that this is not caused by strain variability but by unknown host factors [31,34]. Strain variability, however, has never been tested by cross-passages between ovine VRQ and ovine ARQ transgenic mice. We show that a single, identical prion strain is isolated in both mouse lines, contributing to the evidence that AS is caused by a single prion strain and does not seem to consist of a mixture of strains.

We found no PrP^Sc^ in the lymphoid tissues of the two Dutch sheep with AS. This is in accordance with the findings of many others who failed to detect PrP^Sc^/PrP^res^ with ELISA, WB, IHC or PET (paraffin embedded tissue) blot techniques [6,35, 36]. Andreoletti *et al*. [35] have reported bio-assays in ovine VRQ transgenic mice in order to check for infectivity in lymphoid tissues of sheep with AS. Despite the absence of detectable PrP^Sc^, they found infectivity in the lymphoid tissues of 5/7 sheep with AS. We could not detect any infectivity in the tonsils of the two AS-affected sheep in our study using the ovine VRQ transgenic mice, even though the mice were observed during their entire life span, up to 900 days.

Huor *et al*. [37] reported on the isolation of classical bovine spongiform encephalopathy (c-BSE) from six out of eight European cases of AS in bovine PrP transgenic mice. Three of their transmissions into Tg110 mice were initially negative at primary passage, but c-BSE emerged at secondary passage. We did not see any positive transmission from AS cases to Tg110 mice, neither at primary passage nor at secondary passage, so we cannot confirm the presence of c-BSE in the brain of these two Dutch sheep with AS. If c-BSE were to be co-present with AS, there is no explanation yet for why it would not have emerged naturally from sheep in the field, since AS sheep do carry the genotypes that are also highly susceptible to c-BSE (with relatively short IPs). Since most cases of AS in sheep involve sheep of high age, it can be expected that even low titres of c-BSE prions present would have been able to outgrow AS prions in the brain, especially since the replication sites in the brain are very different. However, to our knowledge, there have been no reports in the literature on AS sheep with mixed PrP^res^ or PrP^Sc^ profiles of both c-BSE and AS in the brain. *In vitro* analysis by Canoyra *et al*. [38] suggested that the emergence of c-BSE could have been caused by a mutation of AS into c-BSE upon transmission to Tg110 mice. In our study, transmissions of ovine AS, as well as transmissions of AS from ovine PrP transgenic mice to Tg110 mice, were all negative, indicating that *in vivo* no mutation of AS into c-type BSE was seen in Tg110 mice. However, we cannot exclude that *in vitro* amplification methods such as Protein Misfolding Cyclic Amplification (PMCA) or Real-Time Quaking-Induced Conversion (RT-QuIC) could be more sensitive to detect c-BSE compared with the *in vivo* bio-assay in bovine transgenic mice.

We did find co-existence of CS in the brain of the AS1 sheep carrying the ALHQ/ALHQ genotype. It is very unlikely that this presence of CS in the AS1 sheep was due to laboratory contamination. The brain material of the sheep used to prepare the inoculum was not taken from the initial brain sample that was sent in for rapid scrapie testing. Instead, we recovered the whole heads of the sheep, aseptically removed the top of the skull and directly sampled the untouched cerebellum with TSE-sterile instruments. Homogenizing rotors to prepare the brain homogenate for mouse bio-assay were also decontaminated (twice) before use, according to proven TSE inactivation procedures. In addition, no TSE cross-contamination has ever been detected in bio-assays of negative control brain tissue homogenates prepared in the same way.

Co-existence of atypical and CS has also been reported by others; however, no previous attempts have been made to strain-type the CS strain [33,39]. We detected in 8% of the transmissions of AS1 to Tg338 mice carrying the ovine VRQ allele, a minor scrapie component with the properties of the CS strain 22A. However, this low-titre component was not detected in the transmissions to the RIII and VM mice carrying the mouse PrP gene, despite the long life span of these mice. This could be due to a higher sensitivity of the Tg338 mice in this bio-assay, due to both a lack of a species barrier (although a genotype barrier may still be present) and the increased level of PrP^C^ in the brain of Tg338 mice, which express the ovine PrP^C^ ~eightfold compared with the levels in the natural host.

22A has previously been reported to have long IPs, exceeding over 5 years after oral inoculation in Cheviot or Suffolk sheep of the ARQ/ARQ genotype [40]. Therefore, it is not surprising that in older sheep with AS, a CS strain can co-exist, which has low virulence for sheep of this genotype. Any classical strain with high virulence toward ARQ/ARQ sheep would most likely have resulted in the succumbing of the animal to CS before AS could ever have emerged.

## Supplementary material

10.1099/jgv.0.002202 Uncited Fig. S1.

## Abbreviations

aco: anterior commissure
alv: alveus
AR: attack rate
AS: atypical scrapie
BSE: Bovine spongiform encephalopathy
c-BSE: classical BSE
cc: corpus callosum
cing: cingulum
CP: caudoputamen
CS: classical scrapie
CTX: cerebral cortex layer
DAB+: diaminobenzidine
dhc: dorsal hippocampal commissure
ec: external capsule
i.c.: intracerebral
IHC: immunohistochemistry
IP: incubation period
PrP: prion protein
PrP^res^: protease-resistant prion protein
PrP^Sc^: scrapie-associated prion protein
PVDF: polyvinylidene difluoride
RT: room temperature
SSBP/1: sheep scrapie brain pool
TBST: Tris buffered saline tween
TSEs: transmissible spongiform encephalopathies
WB: western blot
WBVR: Wageningen bioveterinary research
