# Supplementary material for: Co-presence of classical scrapie but not classical Bovine Spongiform Encephalopathy in transmissions from Dutch sheep with atypical scrapie
Source: J Gen Virol. 2025 Dec 23;106(12):002202. doi: 10.1099/jgv.0.002202 (PMC12723909; doi:10.1099/jgv.0.002202)
Supplement: Uncited Fig. S1. [file jgv-106-02202-s001.pdf]

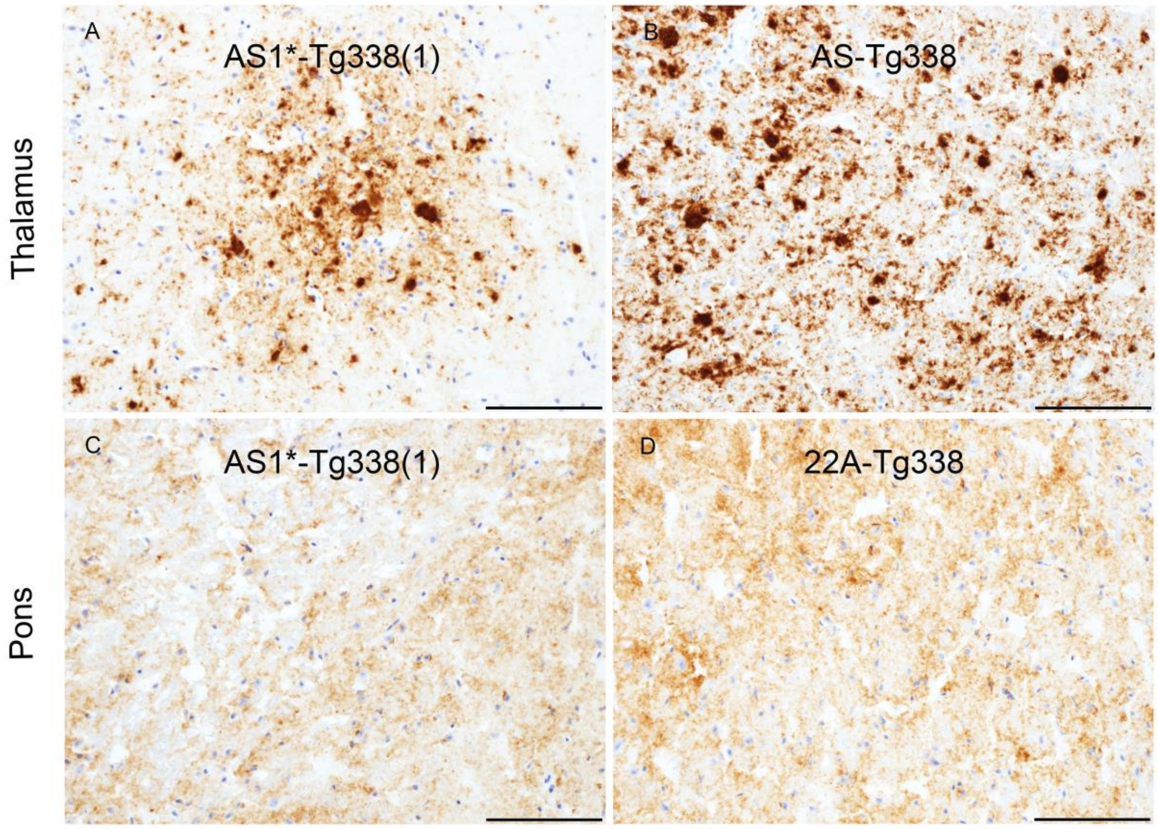

Fig. S1. Comparison of the immunohistochemical PrP^{Sc} patterns of the primary passage of AS1* in Tg338 mice with that of AS and 22A. The PrP^{Sc} staining of AS1* in the thalamus (A) is coarse granular to coalescing with the formation of plaques comparable to the staining of AS in the thalamus (B). However, the PrP^{Sc} staining of AS1* in the pons region (C) is fine granular and only slight coalescing without any visible plaques similar to the staining pattern of 22A in Tg338 mice (D). Bar = 100 µm.
